# Deficiency in Neuroserpin Exacerbates CoCl_2_ Induced Hypoxic Injury in the Zebrafish Model by Increased Oxidative Stress

**DOI:** 10.3389/fphar.2021.632662

**Published:** 2021-03-02

**Authors:** Sha Han, Dongyang Zhang, Qiang Dong, Xu Wang, Liang Wang

**Affiliations:** ^1^ Department of Neurology, Huashan Hospital, Fudan University, Shanghai, China; ^2^ Cancer Metabolism Laboratory, Cancer Research Institute Fudan University Shanghai Cancer Center, Shanghai, China

**Keywords:** neuroserpin, cobalt chloride (CoCl2), hypoxia, protective, oxidative stress

## Abstract

Protective strategy against hypoxic-ischemic (H/I) induced injury has been intensively discussed. Neuroserpin, an inhibitor for tissue plasminogen activator (tPA), has been proved a vital neuroprotective agent in cerebral ischemia mouse model and oxygen-glucose deprivation and reoxygenation (OGD/R) cell model. Neuroserpin is a promising therapeutic hint for neonatal hypoxic-ischemia injury. Here, we established a neuroserpin deficient zebrafish to study its role in CoCl_2_ chemically induced hypoxic injury. CoCl_2_ exposure was beginning at the embryonic stage. Development defects, neuronal loss, and vascular malformation was assessed by imaging microscopy. Neuroserpin deficient zebrafish showed more development defects, neuronal loss and vascular malformation compared to wide-type. Apoptosis and oxidative stress were evaluated to further identify the possible mechanisms. These findings indicate that neuroserpin could protective against CoCl_2_ induced hypoxic injury by alleviating oxidative stress.

## Introduction

Hypoxic stress is involved in not only physiological processes but also pathophysiological conditions such as ischemic stroke, cardiac ischemia, neonatal hypoxic-ischemic (H/I) encephalopathy, and cancer (Hajizadeh et al., 2019; Chen et al., 2020; Miguel and Silveira, 2020; Severino et al., 2020). Normal neuron function is highly dependent on sufficient oxygen supply. Under prolonged hypoxic-ischemic stress, neuronal cells undergo cell death, result in functional impairment, disability, and mortality. Neuroserpin, a tissue plasminogen activator (tPA) inhibitor, has been reported to exert neuroprotective effects in cerebral ischemic and hemorrhagic stroke mouse model (Yepes et al., 2000; Cinelli et al., 2001; Zhang et al., 2002; Lebeurrier et al., 2005; Wu et al., 2010; Rodriguez Gonzalez et al., 2011b; Gelderblom et al., 2013; Li et al., 2017) and stroke patients (Rodriguez Gonzalez et al., 2011c; Wu et al., 2017). *In vitro*, neuroserpin prevents neurons and astrocytes from oxygen-glucose deprivation and reoxygenation (OGD/R) induced injury (Lebeurrier et al., 2005; Rodriguez Gonzalez et al., 2011a; Wang et al., 2015). Related mechanisms involved in inhibiting tPA induced N-methyl-D-aspartic acid neurotoxicity to reduce neuron apoptosis, alleviating inflammatory activities, and preserving blood-brain barrier functions (Yepes et al., 2000; Zhang et al., 2002; Gelderblom et al., 2013; Li et al., 2017). Due to the surging interest in the protective role of plasminogen activator inhibitors, neuroserpin has been suggested as a therapeutic hint in neonatal hypoxia-ischemia mouse model and *ex vivo* (Ma et al., 2012; Millar et al., 2017). We hypothesized that neuroserpin has protective efficacy in a cobalt chloride (CoCl_2_) induced hypoxic injury in a zebrafish model.

In our study, hypoxia stress was induced by CoCl_2_ in zebrafish. CoCl_2_ mimics hypoxia by preventing prolyl and asparaginyl hydroxylase activity and proteasome degradation of the hypoxia inducible factor-1α (HIF-1α) under normoxic conditions, which is the most commonly used method in cells (Kamei and Duan, 2018; Munoz Sanchez and Chanez Cardenas, 2019). The most common methods to study effects of hypoxia in zebrafish during development is the use of CoCl_2_ in the growth medium or incubation of the embryos in a hypoxic chamber with less than 5% O_2_ concentration. However, zebrafish embryos are relatively tolerant to low oxygen (Mendelsohn et al., 2008; Elks et al., 2015). So, CoCl_2_ induced chemical hypoxia injury is more reliable and economic than physical hypoxia chamber in this study.

Zebrafish have several advantages as a chemically induced hypoxia model. Firstly, hypoxia could be induced as early as embryonic stage due to its external development, which makes it possible to observe the phenotypic changes in the developing stage. External embryonic development in zebrafish also enable to study the effects of hypoxia *in vivo* easily and directly. Secondly, CoCl_2_ hypoxia induction is more material and time economical compared to the hypoxia chamber method by addition of drugs direct to the embryo media (Elks et al., 2015). Thirdly, the small size of zebrafish embryos allows high-throughput behavioral screening at a time, minimizing deviation due to temporal and spatial differences.

To verify whether neuroserpin show protective effect on CoCl_2_ induced injury in the zebrafish model, developmental morphological defects, behavioral change, neural lesion, and vascular damage were evaluated in a neuroserpin-deficient zebrafish model. Apoptosis and oxidative stress were assessed to identify related mechanisms. We demonstrate for the first time that neuroserpin exerts a protective role in CoCl_2_ induced hypoxic injury in zebrafish by influencing oxidative stress induced apoptosis.

## Materials and Methods

### Zebrafish Maintenance

Zebrafish (*Danio rerio*) including wild type (WT), Tg (*Huc*: RFP) and Tg (*Fli1*: EGFP) were from the key laboratory of metabolism and molecular medicine, basic medical sciences, Fudan University. Zebrafish were raised and maintained in 14 h light/10 h dark cycle in a standard circulating laboratory environment (28.5°C). All zebrafish related procedures were approved by the Fudan University Shanghai Medical School Animal Care and Use Committee and were conducted in conformity with the National Institutes of Health Guidelines for the Care and Use of Laboratory Animals.

### Establishment of Neuroserpin-Deficient Zebrafish

Neuroserpin-deficient mutant was generated via CRISPR/Cas9 technology as described previously (Hwang et al., 2013). The 19 bp target site 5′-GGC​AAG​AGG​AAC​CTC​CTG​A-3′ of the CRISPR/Cas9 system was designed at the fourth exon of *serpini1* (NC_007129.7) that encodes neuroserpin. The mixture of guide RNA (30 ng/ul) and Cas9 mRNA (300 ng/ul) was injected directly into 1-cell–stage embryos. The genomic DNA of injected embryos at 24 h post-fertilization (hpf) was extracted by DNA lysis buffer and was subjected to PCR amplification. A 386 bp DNA fragment containing the s*erpini1* target site was amplified by PCR (forward primer: TCT​GTG​TTG​TTT​GTG​CTC​AGG, reverse primer: TGC​TGC​AAA​CAT​TAA​CAC​TGC). DNA sequencing was conducted to confirm the mutagenesis. The mutant site was verified by comparison to the WT sequence. Zebrafish that carry the mutation were mated with WT for three generations to obtain *seprini1* ^*+/−*^ heterozygous zebrafish *serpini1* ^*+/-*^. We crossed *serpini1* ^*+/-*^ males and serpini*1* ^*+/-*^ females to obtain *serpini1* ^*−/−*^. To examine the histological change of neuron and vascular, *serpini1* ^*−/−*^ was crossed with Tg (*Huc*: RFP) and Tg (*Fli1*: EGFP) to obtain the Tg (*serpini1* ^*+/-*^-*HuC*: RFP^+/−^) and Tg (*serpini1* ^*+/-*^-*Fli1*: EGFP^+/−^). Tg (*serpini1* ^*−/−*^-*HuC*: RFP^+/+^) and Tg (*serpini1* ^*−/−*^ -*Fli1*: EGFP^+/+^) zebrafish were generated by heterozygote crossing.

### Real-Time PCR

Total RNA was extracted from zebrafish larvae at 7 days post-fertilization (dpf) (n = 30 per sample, three samples per group) using TRIZOL. Primescript RT reagent kit (TAKARA, RR037A, Japan) was used to synthesize cDNA. Real-time PCR was conducted using SYBR Premix Ex Taq II (Takara, RR 420D, Japan) in the StepONEPlus Real-time PCR system (Applied Biosystems, United States). The mRNA levels were normalized to the actin level. The primers for *serpini1* are forward primer- TGA​CGG​CTC​AGA​TGA​CAG​AC; reverse primer- TCC​TGG​TCA​ATG​CGA​TCA​TA.

### CoCl_2_ Induced Hypoxia Injury

Cobalt (II) chloride hexahydrate (CoCl_2_ 6H_2_O, C8661, Sigma, United States) was dissolved in a 100 mM stock solution with sterilized water and stored at −80°C freezer. To find out the preferred concentration for hypoxic injury, different concentrations of CoCl_2_ solution (0, 1, 10, 20, and 50 mM) were applied to zebrafish embryos every 12 h until 96 hpf. Survival rate was calculated under different concentration working solution every 12 h. WT and *serpini1* ^*−/−*^ zebrafish were exposed to the preferred concentration for phenotype observation (survival rate≥50% at 96 hpf) according to the survival curve. Morphology phenotypes including hatch rate at 48hpf, survival rate at 96 hpf, teratogenic effects including pericardial edema, spine deformation, and abnormality of the head, eye (n = 200 per group) were analyzed by visual assessment under a dissection microscope (Olympus, DP73, Japan).

### Behavior

To study the spontaneous activities of WT and *serpini1* ^*−/−*^, a 40-minute behavioral test (10-minute acclimation period with illumination and 30-minute continuous illumination period) was performed at 5 dpf. The detection and recording of zebrafish larvae locomotor activities were achieved by using the tracking mode of ZebraLab software (ViewPoint Life Sciences, France). Videos of zebrafish larvae were taken at the rate of 25 fps (frames per second) and were pooled into 1-minute time bins. The detection threshold was set at 25, a level that allowed the software to accurately detect the movement of the larvae. The threshold for inactive (no locomotion activity) was set to 0 cm/s. General locomotor activities were recorded and analyzed according to the distance moved by the zebrafish larvae in the wells of the 24-well plate (n = 96 per group).

### Imaging Acquisition and Processing

For imaging, live zebrafish larvae anesthetized with 200 mg/L tricaine (Sigma-Aldrich, A5040, United States) were mounted in 3% methylcellulose (Sigma-Aldrich, M7027, United States). Brains were dissected under a dissection microscope (Olympus, DP73, Japan) at the time point of 4 dpf. Fixed samples were balanced and located in 80% glycerol before imaging. Images of neurons in the diencephalon area of Tg (*Huc*: RFP) and Tg (*serpini1* ^*−/−*^ *- HuC*: RFP^+/+^), and vascular of Tg (*Fli1*: EGFP) and Tg (*serpini1* ^*−/−*^ *- Fli1*: EGFP^+/+^) were acquired from a confocal microscope system (Olympus, FV3000, Japan). Using the Z stack strategy, top and bottom of the structure of interest were verified and a 2 um interval was selected to obtain the high-resolution images. Image procession and intensity measurements were done using ImageJ software (n = 10 for each group).

### Acridine Orange Staining

To compare the apoptotic cell numbers of each group, zebrafish larvae at 4 dpf were incubated in 10 μg/ml of AO (Thermo Fisher, A1301, United States) in the culture medium. Phenylthiourea (PTU) treatment can be applied to reduce pigmentation after 24 hpf. After 30 min of staining, larvae were washed three times in culture medium. Larvae were then transferred to 48-well plates and anesthetized with tricaine for imaging (Corning, catalog #4520, Corning, NY). Images were taken in GFP channels (n = 25 per group).

### Oxidative Stress Level Measurements

The content of lipid peroxidation (MDA) was measured by lipid peroxidation assay (Beyotime Institute of Biotechnology, Changsha, China S0131) according to the protocol from the manufacture. 100 larvae from each group were homogenized in ice-cold physiological saline by 1 ml springe and homogenates were centrifuged at 3,000 × g at 4°C for 15 min. Then the supernatants were collected and the assay was carried out immediately. For MDA detection, the supernatants from samples and standard substances were reacted with thiobarbituric acid contained in the kit, and the reaction products were measured spectrophotometrically at 532 nm. Finally, the MDA levels of the samples was calculated according to the standard curve. And the MDA level of samples were normalized against total protein levels determined by the BCA Protein Assay Kit (Beyotime Institute of Biotechnology).

Antioxidant enzyme activity assay: glutathione peroxidase (GPX) (Glutathione peroxidase activity assay: Beyotime Institute of Biotechnology, Changsha, China S0056), catalase (CAT) (Catalase assay: Beyotime Institute of Biotechnology, Changsha, China S0051), and superoxide dismutase (SOD) (Superoxide dismutase assay: Beyotime Institute of Biotechnology, Changsha, China S0101) activity were measured using commercial kits according to the manufacturer’s instructions. Samples from 100 larvae for each group were homogenized and collected. The determination of GPx activity was by detecting reduced NADPH in absorbance at 340 nm. For CAT, different concentrations of hydrogen peroxide (H_2_O_2_) are catalyzed by peroxidase in the kit to produce the red product, N-4-antipyry l-3-chloro-5-sulfonate-p-benzoquinonemonoimine in absorbance at 340 nm. Then, a standard curve for H_2_O_2_ concentration and absorption value was generated. Samples were treated with 250 mM hydrogen peroxide (H_2_O_2_) for 1–5 min. And the remaining H_2_O_2_ was measured at 520 nm. CAT could be calculated according to the standard curve. SOD inhibited the process of superoxide transforming WST-8 to a stable water-soluble WST-8 formazan, which could be tested in the optical density at 450 nm. So, the level of SOD in samples could be calculated according to the A450 absorption. Protein concentration for each sample was quantified by a protein assay kit (Beyotime Institute of Biotechnology). All enzyme activities were normalized as U (unit) per mg of protein.

### Statistical Analysis

Statistical analyses were performed using GraphPad Prism 6 software. Data are expressed as mean ± SEM. Survival rates difference in WT zebrafish under different concentration of CoCl_2_ at the same timepoint were evaluated by one-way ANOVA followed by Dunnett’s multiple comparisons. Statistical analysis for *serpini1* mRNA level, developmental defects, locomotion, neuron loss, and vessel malformation, and oxidative stress level in WT and *serpini1* ^*−/−*^ group under CoCl_2_ induced injury were assessed using unpaired *t*-test. Differences were considered as statistically significant as *p*-value < 0.05.

## Result

### Establishment of Neuroserpin-Deficient Zebrafish Model

**FIGURE 1:**
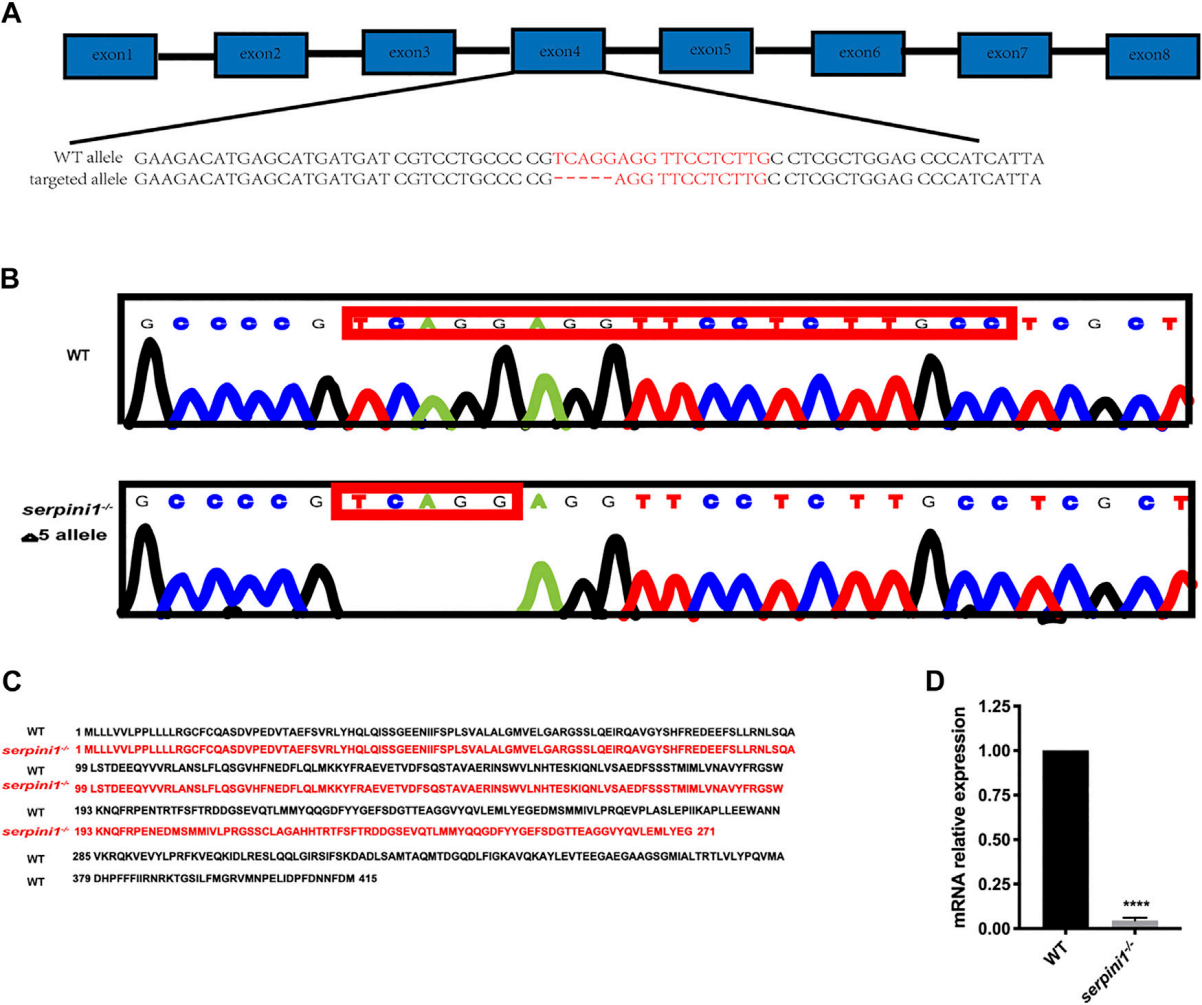
Generation of *serpini1* ^*−/−*^ zebrafish via CRISPR-CAS9 technique. **(A)**: Schematic representation of CRISPR/Cas9 target sites in zebrafish gene *serpini1*. The 19 bp target site is located on the exon4 of the *serpini1* gene. **(B)**: DNA sequencing results of the wild-type (WT) and homozygote *serpini1* ^−/−^. *Serpini1* ^−/−^ mutation leads to five-nucleotide deletion in the fourth exon. **(C)**: Protein comparison for WT and *serpini1* ^−/−^ mutation. The *serpini1* ^−/−^ caused a frameshift mutation, truncated from 415 aa into a 271 aa protein. **(D)**. mRNA level of *serpini1* in *serpini1* ^−/−^ compared to WT. The data are presented as the mean ± SEM, N = 3 trails. Statistical analyses were performed with Student’s *t*-test, ****p < 0.0001 compared to WT.

The *serpini1* ^*−/−*^ mutant zebrafish were successfully generated via CRISPR/Cas9 technique targeting in the fourth exon of the serpini1 sequence (Figure 1A). The DNA sequencing of target-specific PCR products confirmed that the *serpini1* targeted allele caused a frameshift mutation with deletion of five bases (Figure 1B). The mutated variant resulted in a truncated protein with 271 amino acids, which disrupted protein secondary structure (Figure 1C). s*erpini1* mRNA expression was significantly reduced in the *serpini1* ^*−/−*^ zebrafish compared with the WT (Figure 1D). Neuroserpin protein could not be detected due to lack of anti-neuroserpin antibody for zebrafish in the present.

### More Severe Developmental Defects under the CoCl_2_ Induced Hypoxic Injury in Neuroserpin Deficient Zebrafish

**FIGURE 2:**
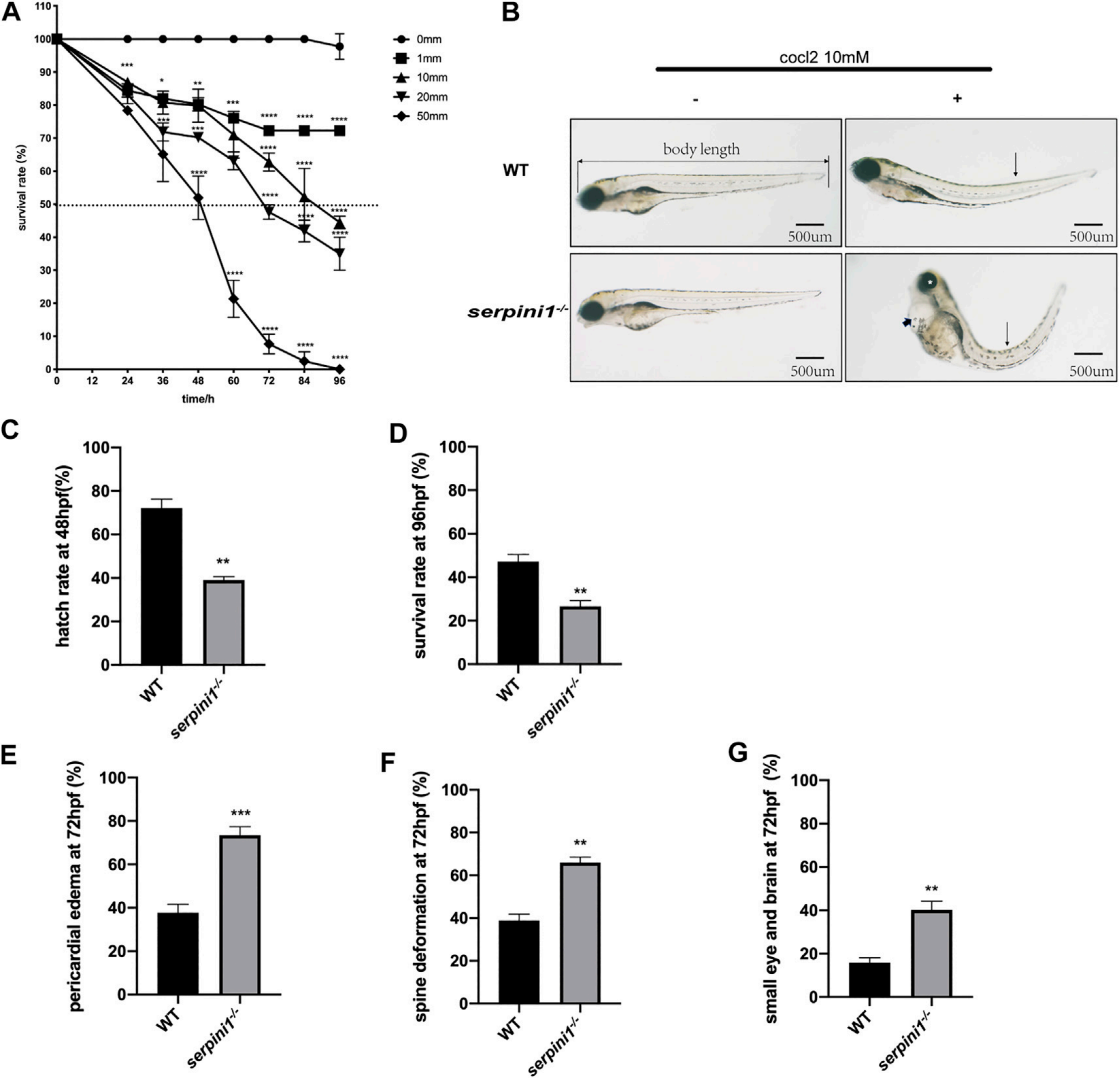
The developmental morphology defects of WT and *serpini1* ^*−/−*^ zebrafish under CoCl_2_ induced hypoxia damage. **(A)** Survival rates of WT under the different concentration of CoCl_2_: 0, 1, 10, 20, 50 mM from 12 hpf to 96 hpf. The data are presented as the mean ± SEM, n = 200 per group. Statistical analyses were performed with one-way ANOVA, followed by Dunnett’s multiple comparisons, *p < 0.05, **p < 0.01, ***p < 0.001, ****p < 0.0001 compared to WT at the same timepoint. Dash line indicates of 50% survival rate. **(B)** Typical morphological defects under the treatment of 10 mM CoCl_2_ in WT and *serpini1* ^*−/−*^ zebrafish at 4dpf; short arrow indicates pericardial edema; long arrow shows spine deformation and asterisk shows small eye deformation. **(C)** Hatch rate at 48 hpf under the treatment of 10 mM CoCl_2_ between WT and *serpini1* ^*−/−*^ group. The data are presented as the mean ± SEM, n = 200 per group; three trails for the experiment. Statistical analyses were performed with Student’s *t*-test, **p < 0.01 compared to WT. **(D)** Survival rate at 96 hpf under the treatment of 10 mM CoCl_2_ between WT and *serpini1* ^*−/−*^ group. The data are presented as the mean ± SEM, n = 200 per group; three trails for the experiment. Statistical analyses were performed with Student’s *t*-test, **p < 0.01 compared to WT. **(E–G)** Percentage of pericardial edema, spine deformation, and small eye and brain deformation in WT and *serpini1* ^*−/−*^ group under the treatment of 10 mM CoCl_2_. The data are presented as the mean ± SEM, n = 200 per group; three trails for the experiment. Statistical analyses were performed with Student’s *t*-test, **p < 0.01, ***p < 0.001.

A series range concentration of CoCl_2_: 0, 1, 10, 20, and 50 mM was used to induce hypoxic injury in WT zebrafish. Survival rates were calculated every 12 h from 12 hpf until 96 hpf. The survival curve showed a time and dose-dependent embryotoxicity (Figure 2A). Almost all embryos that subjected to 50 mM CoCl_2_ group were dead after exposure for 96 hpf. 96 hpf was selected as the timepoint for morphological experiments because zebrafish accomplish development at this time. 10 mM CoCl_2_, which caused 50% death in the WT group, was a selective concentration to study the effect of neuroserpin on morphological defects under CoCl_2_ induced hypoxic injury. As shown in Figure 2B, 10 mM CoCl_2_ caused developmental abnormalities including pericardial edema, spine deformation, and eye and brain deformation at 96 hpf. Compared to the WT, *serpini1* ^*−/−*^ group had decreased hatch rate at 48 hpf (WT vs. *serpini1* ^*−/−*^: 72.1 ± 4.085 vs. 39 ± 1.595) (Figure 2C) and reduced survival rate at 96hpf (WT vs *serpini1* ^*−/−*^: 47.13 ± 3.323 vs. 26.63 ± 2.621) (Figure 2D). Moreover, *serpini1* ^*−/−*^ group increased the rate of pericardial edema (WT vs. *serpini1* ^*−/−*^: 37.77 ± 2.233 vs. 73.47 ± 2.256), spine curvature (WT vs. *serpini1* ^*−/−*^: 38.9 ± 2.967 vs. 65.93 ± 2.598), and small brain and eye deformation (WT vs *serpini1* ^*−/−*^: 15.87 ± 2.248 vs. 40.17 ± 4.013) compared to WT at the time point of 96 hpf (Figures 2F,G). These results indicated that neuroserpin-deficient zebrafish showed more severe developmental defects.

### Increased Locomotor Impairment and More Neuron Loss in Neuroserpin Deficient Zebrafish under CoCl_2_ Induced Hypoxic Injury

**FIGURE 3:**
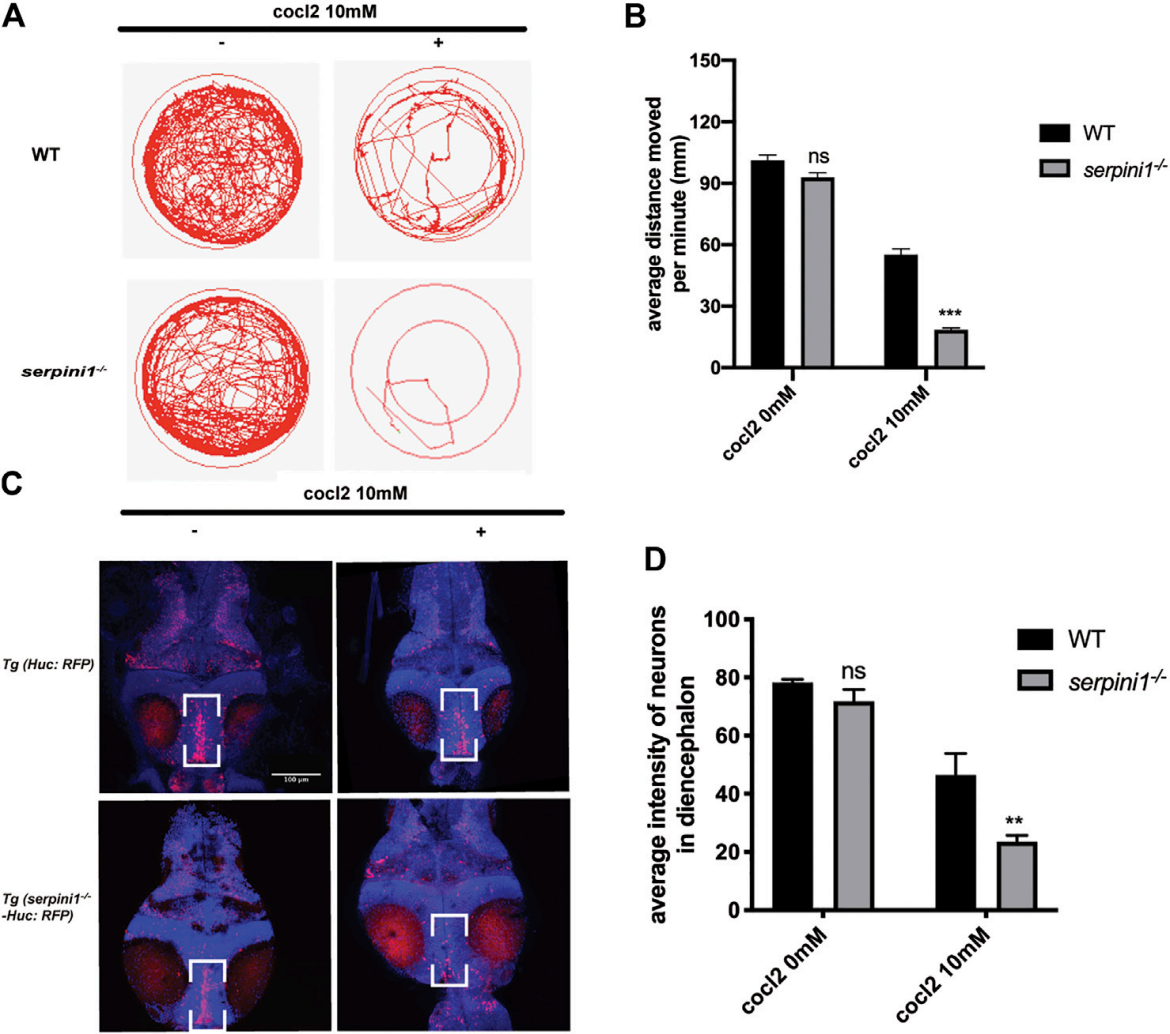
Neuroserpin deficient zebrafish showed reduced locomotor activities and more neurons loss in diencephalon area under CoCl_2_ induced hypoxic injury. **(A)** Representative graph for distance traveled in 40 min for WT and *serpini1* ^*−/−*^ zebrafish. **(B)** Quantification of average distance moved per minute in WT and *serpini1* ^*−/−*^ under 10 mM CoCl_2_ induced injury. The data are presented as the mean ± SEM, n = 96 per group. Statistical analyses were performed with Student’s *t*-test, ***p < 0.001, ns: no significant, compared with WT in the same CoCl_2_ concentration. **(C)** Images of neurons in diencephalon in Tg (*Huc*: RFP) and Tg (*serpini1* ^*−/−*^-*HuC*: RFP) under 10 mM CoCl_2_ induced injury. Scale bar:100 um. **(D)** Quantification of average fluorescence intensity of neurons in diencephalon area in Tg (*Huc*: RFP) and Tg (*serpini1* ^*−/−*^-*HuC*: RFP) under 10 mM CoCl_2_ induced injury. The data are presented as the mean ± SEM, n = 6 per group. Statistical analyses were performed with Student’s *t*-test, **p < 0.01, ns: no significant, compared with WT in the same CoCl_2_ concentration.

To further examine the behavioral change under CoCl_2_ induced hypoxia injury, 5 dpf zebrafish from WT and *serpini1* ^*−/−*^ were incubated in E3 medium in 24-plate and general locomotor movement was recorded. CoCl_2_ induced hypoxic injury caused decreased locomotor activities in both groups as shown in Figures 3A,B (WT: 0 mM CoCl_2_ vs. 10 mM CoCl_2:_ 101.2 ± 2.60 vs. 55.17 ± 2.18) (*serpini1* ^*−/−*^: 0 mM CoCl_2_ vs. 10 mM CoCl_2:_ 92.94 ± 2.23 vs. 18.47 ± 0.88). However, the average distance moved per minute by *serpini1* ^*−/−*^ decreased significantly compared to WT under 10 mM CoCl_2_ induced hypoxia injury (WT vs *serpini1* ^*−/−*^: 55.17 ± 2.18 vs. 18.47 ± 0.88) (Figure 3B). Brain lesion was assessed by analyzing the neurons in the diencephalon area in Tg (*Huc*: RFP) and Tg (*serpini1* ^*−/−*^-*HuC*: RFP) at 4dpf. Tg (*serpini1* ^*−/−*^-*HuC*: RFP) did not show difference in red fluorescence intensity of neurons in diencephalon area compared to Tg (*Huc*: RFP) (WT vs. *serpini1* ^*−/−*^: 78.26 ± 1.06 vs. 71.79 ± 2.34) (Figure 3D). However, the average red fluorescence intensity values in Tg (*serpini1* ^*−/−*^-*HuC*: RFP) group under CoCl_2_ induced hypoxia injury were comparatively lower than that in Tg (*Huc*: RFP) (WT vs. *serpini1* ^*−/−*^: 46.46 ± 4.27 vs. 24.54 ± 1.25), suggesting that more neuron loss in Tg (*serpini1* ^*−/−*^ *-HuC*: RFP) group in the diencephalon area under 10 mM CoCl_2_ injury (Figure 3D).

### Aggravated Vascular Malformation in Neuroserpin Deficient Zebrafish under CoCl_2_ Induced Hypoxic Injury

**FIGURE 4:**
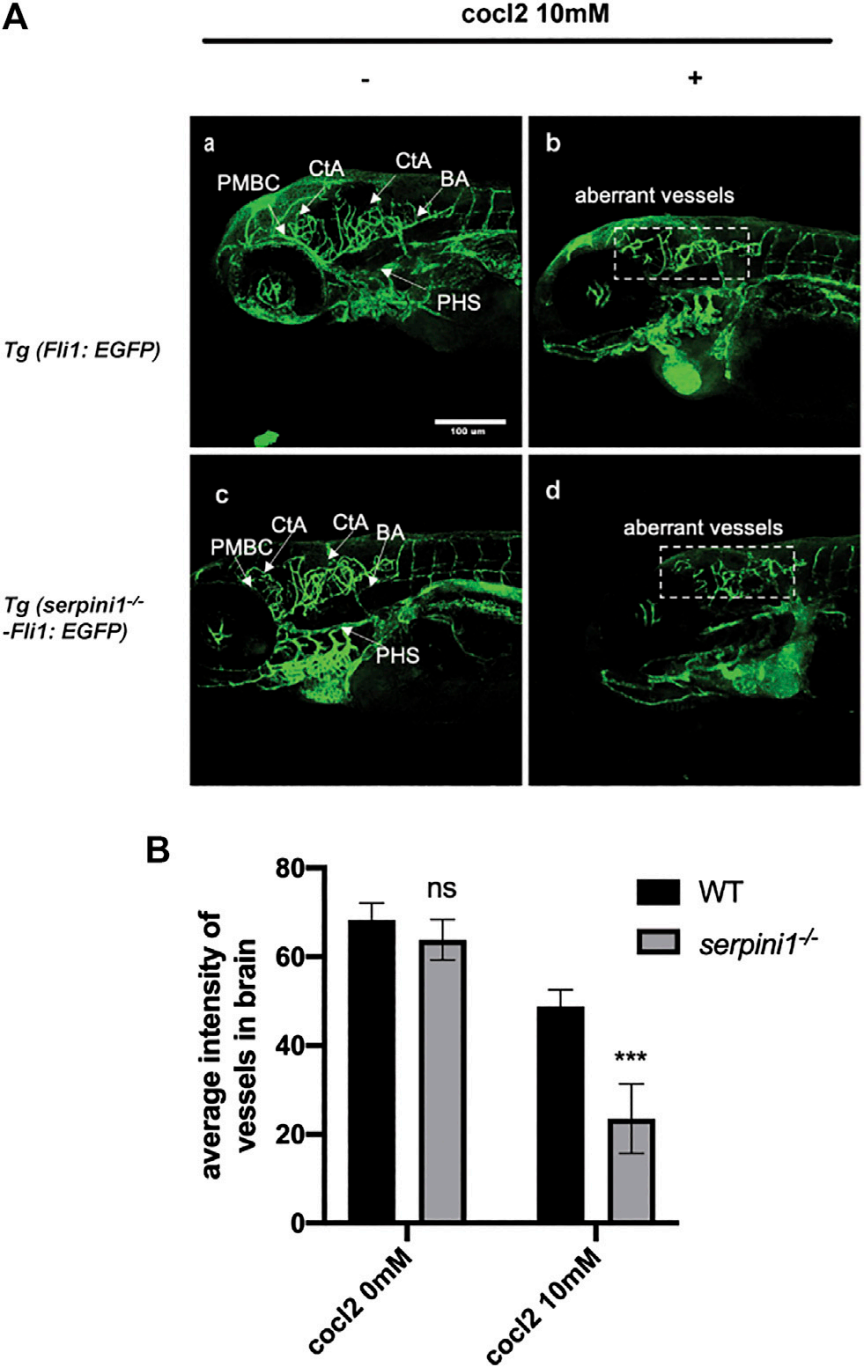
More severe vascular malformation in neuroserpin deficient zebrafish under CoCl_2_ induced hypoxic injury. **(A)** Image of vessels of Tg (*Fli1*: EGFP) and Tg (*serpini1* ^*−/−*^-*Fli1*: EGFP) with or without 10 mM CoCl_2_ induced injury. Tg (*Fli1*: EGFP) and Tg (*serpini1* ^*−/−*^-*Fli1*: EGFP) show normal vascular morphology in brain region including central artery (CtA), primordial hindbrain channel (PHBC), and primary head sinus (PHS). CoCl_2_ caused vascular malformation both in Tg (*Fli1*: EGFP) and Tg (*serpini1* ^*−/−*^-*Fli1*: EGFP) under 10 mM CoCl_2_-induced injury. More reduced vascular branches in brain area were observed in Tg (*serpini1* ^*−/−*^-*Fli1*: EGFP) group compared to Tg (*Fli1*: EGFP). Scale bar:100 um. White arrow indicates brain vessels. Dash white square box shows reduction of normal vessels in CoCl_2_ treated groups. **(B)** Quantification of average fluorescence intensity of vessels in brain area in Tg (*Fli1*: EGFP) and Tg (*serpini1* ^*−/−*^-*Fli1*: EGFP) under 10 mM CoCl_2_ induced injury. The data are presented as the mean ± SEM, n = 6 per group. Statistical analyses were performed with Student’s *t*-test, ***p < 0.001, ns: no significant, compared with WT in the same CoCl_2_ concentration.

The vessel structures in the Tg (*Fli1*: EGFP) and Tg (*serpini1* ^*−/−*^ -*Fli1*: EGFP) groups were normal and the sprouting of blood vessels was visually observed at 96 hpf (Figure 4A,C). Average fluorescence intensity of vessels in the brain region in these two groups were statistically comparable (WT vs *serpini1* ^*−/−*^: 68.26 ± 2.73 vs. 63.79 ± 2.63) (Figure 4B). Vascular malformation was observed in Tg (*Fli1*: EGFP) and Tg (*serpini1* ^*−/−*^ -*Fli1*: EGFP) zebrafish larval under CoCl_2_ induced hypoxic injury. Abundant blood vessels in the brain degenerated in the embryos exposed to CoCl_2_ (Figures 4A,B,D). More reduced normal vessels in the brain area were determined in Tg (*serpini1* ^*−/−*^ -Fli1: EGFP) compared to Tg (*Fli1*: EGFP) under CoCl_2_ induced hypoxic injury (Figure 4A). The decrease in average fluorescence intensity of vessels in the brain region under CoCl_2_ exposure was statistically substantial in Tg (*serpini1* ^*−/−*^ -*Fli1*: EGFP) zebrafish compared to Tg (*Fli1*: EGFP) (WT vs. *serpini1* ^*−/−*^: 48.80 ± 2.15 vs. 23.53 ± 4.53) (Figure 4B).

### Apoptosis and Oxidative Stress Level in Neuroserpin Deficient Zebrafish under CoCl_2_ Induced Hypoxic Injury

**FIGURE 5:**
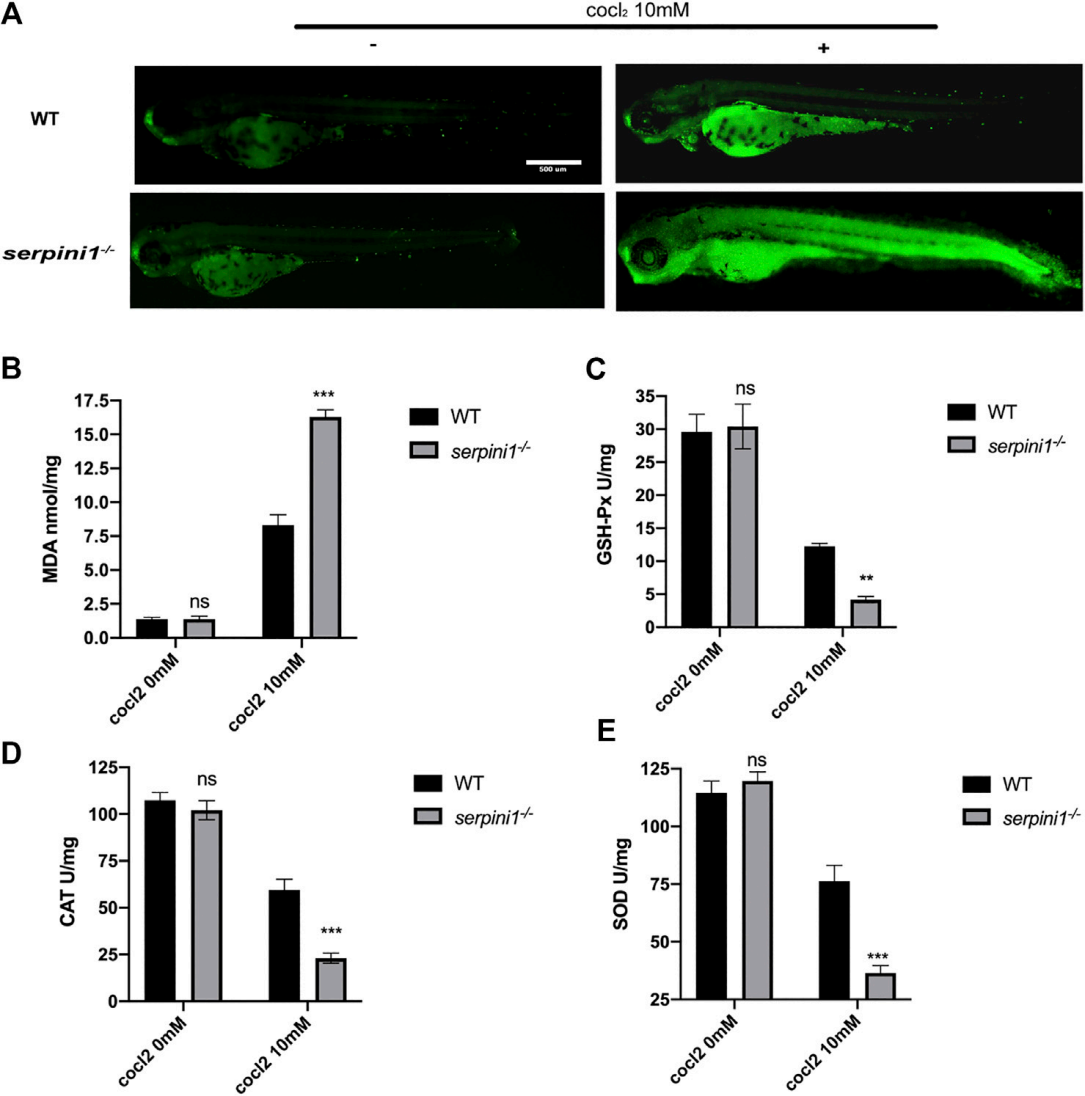
Apoptosis and oxidative stress were more severely enhanced in *serpini1* ^*−/−*^ zebrafish group under CoCl_2_ induced hypoxic injury. **(A)** AO staining of the apoptotic cells in the live image of WT and *serpini1* ^*−/−*^ zebrafish under the treatment of 10 mM CoCl_2_ at 4dpf. Scale bar:500 um. **(B)** MDA content in WT and *serpini1* ^*−/−*^ under 10 mM CoCl_2_ induced injury. The data are presented as the mean ± SEM, n = 100 per group. Three trails for the experiment. Statistical analyses were performed with Student’s *t*-test, ***p < 0.001 compared with WT, ns: no significant. **(C)** The level of GSH-Px in WT and *serpini1* ^*−/−*^ under 10 mM CoCl_2_ induced injury. The data are presented as the mean ± SEM, n = 100 per group. Three trails for the experiment. Statistical analyses were performed with Student’s *t*-test, **p < 0.01 compared with WT, ns: no significant. **(D)** The level of CAT in WT and *serpini1* ^*−/−*^ under 10 mM CoCl_2_ induced injury. The data are presented as the mean ± SEM, n = 100 per group. Three trails for the experiment. Statistical analyses were performed with Student’s *t*-test, ***p < 0.001 compared with WT, ns: no significant. **(E)** The level of SOD in WT and *serpini1* ^*−/−*^ under 10 mM CoCl_2_ induced injury. The data are presented as the mean ± SEM, n = 100 per group. Three trails for the experiment. Statistical analyses were performed with Student’s *t*-test, ***p < 0.001 compared with WT, ns: no significant.

To further evaluate the CoCl_2_ induced injury, AO staining was used to identify the apoptotic cells. 10 mM CoCl_2_ induced gross apoptotic damage in the brain, heart, and spinal cord (Figure 5A). *serpini1* ^*−/−*^ group displayed increased apoptosis compared to WT (Figure 5A). To identify the mechanism, oxidative stress levels were assessed in both groups under 10 mM CoCl_2_ induced injury. As shown in Figures 5B–E), *serpini1* ^*−/−*^ group significantly increased peroxidation product MDA (Figure 5B) (WT vs. *serpini1* ^*−/−*^: 8.301 ± 0.774 vs. 16.290 ± 0.522). In addition, the activities of GPx (WT vs. *serpini1* ^*−/−*^: 12.270 ± 0.442 vs. 4.181 ± 0.463), CAT (WT vs. *serpini1* ^*−/−*^: 156.045 ± 6.285 vs. 219.719 ± 9.585) and SOD (WT vs *serpini1* ^*−/−*^: 176.328 ± 6.826 vs. 243.908 ± 11.716) were significantly decreased in *serpini1* ^*−/−*^ group under 10 mM CoCl_2_ exposure compared to WT (Figures 5C–E) (n = 100 per group per trail, three trials for each experiment).

## Discussion

Research on protective therapy for cerebral ischemia revealed that neuroserpin is an essential therapeutic agent in the pathological condition and gives us a hint that it could protect against neonatal hypoxic ischemia. (Cinelli et al., 2001; Lebeurrier et al., 2005; Rodriguez Gonzalez et al., 2011b; Gelderblom et al., 2013). Due to its high efficacy in gene-manipulation, we establish a neuroserpin knockout zebrafish to study whether neuroserpin inserts protective role in CoCl_2_ induced hypoxia injury in this study.

Moderate to high concentration of CoCl_2_ could cause embryotoxicity and development defects in zebrafish (Cai et al., 2012). In our study, CoCl_2_ was exposed in the embryonic stage. Reduced hatchment, increased mortality and more teratogenic effects were observed in neuroserpin deficient zebrafish group. These developmental defects result in more severe behavioral impairment and neuronal loss in brain. Our study reflects the same phenotypes in neonatal hypoxia brain injury such as developmental retardation, spine deformation, microcephaly and microphthalmia, which causes long-term disability and mortality. Plasminogen activator (PA) system is critical for hypoxic-ischemic brain injury in newborns. Endogenous neuroserpin and tissue plasminogen activator (tPA) highly express at the early development stage in central nervous system and decline to a moderate level at the adult stage, which are involved in neuronal migration, axonal outgrowth, and synapse plasticity (Lee et al., 2017). Recent researches also detected that neuroserpin is expressed in the developing cortical plate and subplate, which is sensitive to neonatal ischemia hypoxia injury (Adorjan et al., 2019; Kondo et al., 2015). tPA induction was deleterious in hypoxic-ischemic Vannucci model of hypoxic-ischemic encephalopathy and in the adult ischemic stroke model (Adhami et al., 2008; Omouendze et al., 2013). So, serine-protease inhibitors could be a protective therapy strategy. It has been shown that plasminogen activator inhibitor-1(PAI-1) by intranasal delivery or ventricle injection protect against neonatal hypoxic-ischemia injury by reduced axonal degeneration and cortical neuron death (Yang et al., 2009; Yang et al., 2013; Yang and Kuan, 2015). As with PAI-1 and other serine-protease inhibitors, neuroserpin shares the essential conserved inhibitory center reactive center loop (RCL) to inhibit tPA activity. These evidences indicate that neuroserpin could be considered as a protective agent for neonatal hypoxic ischemia.

CoCl_2_ provokes HIF-1α intracellular accumulation, which stimulates vascular endothelial growth factor (VEGF) secretion, angiogenesis, and hemorrhages. It has been verified that blood-brain barrier (BBB) is impaired in both ischemic stroke and neonatal hypoxic-ischemia encephalopathy (Abu Fanne et al., 2010; Tu et al., 2011). Excessive tPA is induced under hypoxia stress in microvascular endothelial cells (Henry et al., 2013) and elevated tPA could trigger the opening of BBB through plasmin-dependent pathway or matrix metallopeptidases activation (Su et al., 2008). Our previous data in an intracerebral hemorrhage mouse model identify that neuroserpin restores blood-brain barrier by re-establishing tight junction of vascular endothelial cells. In the present study, aggravated vascular malformation was observed in neuroserpin deficient group and these vessels are fragile and prone to rupture, which may due to dysfunction of vascular endothelial cells. It may highlight the complicated role of neuroserpin in endothelial cells. Neuroserpin has been found to express in non-neuronal cells such as the ependyma and epithelial cells of choroid plexus (Teesalu et al., 2004) and other tissues such as endocrine and immune systems (Fell et al., 2002; Hill et al., 2002). The impact of neuroserpin in non-neuronal cells and tissues may be involved in regulation of cell-cell adhesion or modification of extracellular matrix (Parmar et al., 2002; Lee et al., 2008; Gupta et al., 2017). More evidence is needed to be explored in the future study.

Cobalt-induced hypoxia associated mechanisms of toxicity are not yet understood. Prolonged or high-dose treatment with CoCl_2_ switches cell metabolism from aerobic respiration to anaerobic glycolysis (Regazzetti et al., 2009). This induction of oxidative stress can provoke cell death through apoptosis (Stewart et al., 2017). In our study, we found augmented apoptosis and enhance oxidative stress level in *serpini1* ^*−/−*^ zebrafish under cobalt-induced hypoxia. The generation of excessive oxidative damage is believed to be involved in the pathogenesis of neonatal hypoxic-ischemic injury. A study of extracellular application of recombinant neuroserpin to primary hippocampal culture confirmed that it could rescue hydrogen peroxide (H_2_O_2_)-induced oxidative stress neurotoxicity (Cheng et al., 2017). Our finding was consistent with the result and it could be an essential mechanism for protection role of neuroserpin.

Although we confirmed that neuroserpin protects against CoCl_2_ induced hypoxia injury in the zebrafish model in present study, it should be noted that this study uses a hypoxia-only model without ischemia. Human hypoxic-ischemic injury is usually more complicated with insufficient blood supply due to vascular occlusion or rupture. So, this artificial hypoxia-only system is not ideal for further complex conclusion. In addition, there are many other effects of hypoxia-ischemia that are not mimicked by CoCl_2_ treatments. Hypoxia downregulates HIF-1α during prolonged oxygen reduction. The decrease in HIF-1α protein levels could be mediated by the increase in the levels of the prolyl hydroxylases (Berra et al., 2003) or by the induction of antisense HIF-1α (Rossignol et al., 2002), which can act in a negative manner. However, the negative feedback regulation is not established when HIF-1α is stabilized by CoCl_2_, which affect severely the proliferative capacity of Hela cell (Triantafyllou et al., 2006). So, the phenotypes we observed in our study maybe different from the pathological change in hypoxic-ischemic injury. Furthermore, CoCl_2_ have alternative and more complex mechanisms of cobalt activity. High concentration of CoCl_2_ used to induce hypoxia has been shown to influence hematological cells of fish (Saeedi Saravi et al., 2009). Also, CoCl_2_ is known to cause mitochondrial damage (Mansfield et al., 2005). We may not exclude the off-target effects of CoCl_2_ in our study. These problems deserve further investigation and exploration.

To the best of our knowledge, this is the first report that demonstrates the protection of neuroserpin in zebrafish under CoCl_2_ induced hypoxia injury. This study contributes to a better understanding of the protective role of neuroserpin in hypoxic injury.

## Data Availability

The raw data supporting the conclusions of this article will be made available by the authors, without undue reservation.
